# Costs of Illness Due to Cholera, Costs of Immunization and Cost-Effectiveness of an Oral Cholera Mass Vaccination Campaign in Zanzibar

**DOI:** 10.1371/journal.pntd.0001844

**Published:** 2012-10-04

**Authors:** Christian Schaetti, Mitchell G. Weiss, Said M. Ali, Claire-Lise Chaignat, Ahmed M. Khatib, Rita Reyburn, Radboud J. Duintjer Tebbens, Raymond Hutubessy

**Affiliations:** 1 Department of Epidemiology and Public Health, Swiss Tropical and Public Health Institute, Basel, Switzerland; 2 University of Basel, Basel, Switzerland; 3 Public Health Laboratory Ivo de Carneri, Ministry of Health of Zanzibar, Chake-Chake, Pemba, United Republic of Tanzania; 4 Global Task Force on Cholera Control, World Health Organization, Geneva, Switzerland; 5 Ministry of Health of Zanzibar, Zanzibar, United Republic of Tanzania; 6 International Vaccine Institute, Seoul, Korea; 7 Kid Risk, Inc., Newton, Massachusetts, United States of America; 8 Initiative for Vaccine Research, World Health Organization, Geneva, Switzerland; Massachusetts General Hospital, United States of America

## Abstract

**Background:**

The World Health Organization (WHO) recommends oral cholera vaccines (OCVs) as a supplementary tool to conventional prevention of cholera. Dukoral, a killed whole-cell two-dose OCV, was used in a mass vaccination campaign in 2009 in Zanzibar. Public and private costs of illness (COI) due to endemic cholera and costs of the mass vaccination campaign were estimated to assess the cost-effectiveness of OCV for this particular campaign from both the health care provider and the societal perspective.

**Methodology/Principal Findings:**

Public and private COI were obtained from interviews with local experts, with patients from three outbreaks and from reports and record review. Cost data for the vaccination campaign were collected based on actual expenditure and planned budget data. A static cohort of 50,000 individuals was examined, including herd protection. Primary outcome measures were incremental cost-effectiveness ratios (ICER) per death, per case and per disability-adjusted life-year (DALY) averted. One-way sensitivity and threshold analyses were conducted. The ICER was evaluated with regard to WHO criteria for cost-effectiveness. Base-case ICERs were USD 750,000 per death averted, USD 6,000 per case averted and USD 30,000 per DALY averted, without differences between the health care provider and the societal perspective. Threshold analyses using Shanchol and assuming high incidence and case-fatality rate indicated that the purchase price per course would have to be as low as USD 1.2 to render the mass vaccination campaign cost-effective from a health care provider perspective (societal perspective: USD 1.3).

**Conclusions/Significance:**

Based on empirical and site-specific cost and effectiveness data from Zanzibar, the 2009 mass vaccination campaign was cost-ineffective mainly due to the relatively high OCV purchase price and a relatively low incidence. However, mass vaccination campaigns in Zanzibar to control endemic cholera may meet criteria for cost-effectiveness under certain circumstances, especially in high-incidence areas and at OCV prices below USD 1.3.

## Author Summary

Despite efforts to improve water supply and sanitation, cholera still represents a serious burden in developing countries. Use of oral cholera vaccines (OCVs) in endemic and epidemic situations has recently shown a promising potential to mitigate this burden. To provide local decision-makers with specific information on OCV use for cholera control, we assessed the costs and benefits of a mass vaccination campaign that was conducted in 2009 in selected endemic areas of Zanzibar. We estimated the cost-effectiveness of OCVs by collecting health care provider and household costs of illness from cholera outbreaks and costs of the mass vaccination campaign that used the two-dose OCV Dukoral. Cost-effectiveness was expressed as the incremental costs of the one-off vaccination program per case, per death and per disability-adjusted life-year averted, over a three-year time period. Our model showed that the 2009 mass vaccination campaign in Zanzibar was not cost-effective, mainly due to the high OCV price (USD 10) and the relatively low incidence. Threshold analyses with Shanchol, the second OCV that is recommended by the WHO, indicated that mass vaccination in Zanzibar to control endemic cholera may become cost-effective if done in higher incidence areas and when OCV prices are reduced to levels below USD 1.3.

## Introduction

Despite efforts to improve water supply and sanitation, cholera still represents a serious public health burden in low- and middle-income countries. In 2009, more than 220,000 cases and almost 5,000 deaths were reported to the World Health Organization (WHO) [1]. Due to underreporting and difficulties with surveillance, however, the true burden is likely in the range of 3 million cases and 100,000 deaths per year [2], [3]. A recent review of official cholera-related morbidity and mortality data from the WHO Africa region also indicated a potential economic burden of cholera for families and the health sector [4].

Cholera is an enteric bacterial disease caused by *Vibrio cholerae* serogroup O1 or O139 that usually occurs in sudden epidemics. Main features include acute, profuse watery diarrhea and vomiting that may lead to dehydration with concurrent electrolyte loss and eventually death if timely treatment is unavailable. Even though case-fatality rates (CFRs) may reach 50%, a rate below 1% has been achieved with proper case management [3], [5]. Treatment is based on prompt rehydration with oral rehydration solution (ORS) for mild to moderate cases and intravenous (IV) fluids for severe cases [3]. Antibiotics are recommended for severe, and also moderate cases, to reduce the duration of episodes and shedding of infectious *V. cholerae* [3], [6].

Traditionally, cholera control has been based on prevention (i.e., adequate water supply, improved sanitation and health education, and timely treatment). The role of vaccination for cholera control has recently received increased attention from public health officials; the WHO recommends oral cholera vaccines (OCVs) as a supplementary public health tool to traditional prevention and treatment in endemic and epidemic settings [7].

A series of research studies, done as part of the Diseases of the Most Impoverished (DOMI) project coordinated by the International Vaccine Institute (IVI), evaluated the use of OCVs in Asia and Africa for control of endemic cholera. Private demand for cholera vaccines was examined through willingness-to-pay studies [8]–[11], costs of illness (COI) and mass vaccination data were collected [12]–[14], and cost-effectiveness and cost-benefit analyses were performed [15], [16]. Besides the recent article by Poulos *et al.* [13], published information about COI due to cholera is lacking even though patient-level data is needed for economic evaluations to improve local planning of cholera control.

A joint initiative between the WHO, the IVI and the Ministry of Health of Zanzibar (MoH) implemented a mass vaccination campaign with an OCV in two selected cholera-endemic areas of Zanzibar in 2009. This intervention-*cum*-research project provided the opportunity to assess costs of immunization in an endemic setting. Public COI—defined as fixed and variable costs borne by the health care provider for setting up and running cholera treatment centers (CTCs)—were estimated from three outbreaks that happened in 2009 outside the mass vaccination target communities. Private direct COI—defined as medical and non-medical expenses related to patient treatment, and indirect COI—defined as loss of income borne by patients and their families—were elicited from a sample of patients admitted to CTCs during these outbreaks.

This study aims to estimate (i) public and private COI due to cholera, (ii) costs of an oral cholera mass vaccination campaign, and (iii) the cost-effectiveness of using OCVs in endemic regions of Zanzibar from a health care provider and a societal perspective.

## Methods

### Ethics Statement

Written informed consent was obtained from all study participants interviewed for private costs of illness. Patients aged 18 years or older were directly interviewed while caregivers were interviewed if the patient was younger than 18 years. No incentives were provided to them. The protocol of this study was cleared by the WHO Research Ethics Review Committee and the MoH Ethics Committee. All data were handled confidentially and made anonymous before analysis.

### Study Setting

Zanzibar consists of two major islands, Unguja (also named Zanzibar) and Pemba, which are situated in the Indian Ocean about 40–60 km off the coast of Tanzania. Zanzibar, a semiautonomous polity within the United Republic of Tanzania, consists of five regions, which are subdivided into ten districts, 50 constituencies and 296 Shehias, the latter being the smallest administrative unit. The main islands cover ∼2,557 km^2^ (Unguja: ∼1,651 km^2^, Pemba ∼906 km^2^). The archipelago is inhabited by a fast-growing population of ∼1.2 million Kiswahili-speaking Muslim people. Monthly mean per capita expenditure for all goods and services was TZS 21,000 (∼USD 18) in 2004/5 with a 2.1% share for health-related expenditures [17]. Life expectancy at birth has risen from 47 years in 1988 to 57 years in 2002 [18]. The economy of the islands depends on agriculture (primarily cloves, coconuts/copra and seaweed), fishing and tourism.

The public health care delivery structure in Zanzibar comprises two zones, Unguja and Pemba, each with three levels: the primary, the secondary and the tertiary level. Each zone is headed by a zonal medical officer. Most of the health care services are provided at the primary level through Primary Health Care Units (PHCU) (n = 124). The majority of these units is open during the day to outpatients and provides basic services. Primary Health Care Centers (PHCC) (n = 4) are additional facilities on the primary level; they operate on a 24-hours basis and can admit up to 30 patients. At the secondary level, three district hospitals (only in Pemba) are operational while the country's only tertiary level hospital (Mnazi Mmoja) is located in the capital Stonetown in Unguja. The top causes of primary- and secondary-level outpatient visits in 2008 were upper respiratory tract infections (23%), pneumonia (10%), malaria (10%) and diarrhea (9%) [19].

In recent times, the first cholera outbreak with 411 cases and 51 deaths was reported in 1978 from two fishermen villages in Zanzibar [20]. More than a dozen outbreaks followed since then with almost annual episodes since the year 2000. Reyburn *et al.* reported an annual incidence of 0.5 cases per 1,000 population based on a review of routine surveillance data for the years 1997 to 2007 [21], although the true incidence was likely higher due to underreporting. A seasonal pattern can be observed that follows the rainy seasons (usually from March to June and from October to December) during which widespread flooding occurs. Such deteriorating environmental conditions subsequently expose the majority of inhabitants on both islands to an increased risk of waterborne diseases due to the scarcity of safe drinking water supplies and a generally poor or lacking sanitation infrastructure in periurban and rural areas.

Based on a consideration of areas of recent cholera activity, three Shehias per island, adjacent to each other, were selected as sites for the mass vaccination campaign. In Unguja, the Shehias of Chumbuni and Karakana in Urban district and Mtopepo in West district were targeted for the campaign; in Pemba, the Shehias of Kengeja, Mwambe and Shamiani, all located in the rural southeastern Mkoani district, were chosen.

Dukoral, the only OCV that was pre-qualified by the WHO in 2009, was used in the mass vaccination campaign. Dukoral is a *V. cholerae* serogroup O1 whole-cell, killed vaccine containing recombinant cholera toxin (CT) B subunit protein; it has to be administered in two doses at least one week apart and requires a cold chain (2–8°C) [22]. This OCV was originally designed for immunologically naïve travelers from the north to tropical countries; it is licensed for use from two years of age and above and was shown to be 60–90% protective for up to three years [23]–[25]. One three-ml vial of Dukoral contains 1×10^11^ killed *V. cholerae* O1 (biotype classical and El Tor) and 1 mg of the CT B subunit protein in a suspension. Because the CT B subunit protein is not gastric acid-fast, the suspension has to be mixed with 1.5 dl of drinking water and a buffer sachet containing effervescent granules of sodium bicarbonate before ingestion. Recipients need to fast one hour before and after ingestion.

### Cost Data Collection

Table 1 describes cost components and sources of data collected for this study. Estimates for public COI were obtained from interviews with local experts and unvaccinated patients and from reports and record review. Cost data for the mass vaccination campaign were collected based on actual expenditure and planned budget data. Private direct and indirect costs were collected through interviews done with unvaccinated patients on Pemba. All costs are reported in 2009 USD from an economic perspective, based on mid-2009 exchange rates obtained from http://www.oanda.com/currency/converter/.

**Table 1:** Cost components for cholera collected in Zanzibar, 2009.

Cost components	Description	Source
**Public COI**		
Fixed costs	CTC set up and running including top up payments and personnel opportunity costs	Questionnaire for zonal and district medical officers, MoH, NGOs, reports, record review
Variable costs	Treatment costs including drugs and material	Interview with laboratory-confirmed cases and health care personnel, questionnaire for zonal and district medical officers, chief pharmacist, NGOs
**Private COI**		
Direct	Medical, non-medical costs	Interview with laboratory-confirmed cases
Indirect	Loss of income	Interview with laboratory-confirmed cases
**Mass vaccination campaign costs**		
Material	Purchase, transport and storage of vaccine, water and cups	Reports and documents from WHO HQ, WHO consultants, EPI
WHO consultants	Compensation, travel	Communication from WHO HQ
Training of vaccinators and social mobilizers	Staff compensation, transport, material, refreshment, venue	Reports and documents from WHO consultants, EPI
Implementation	Staff compensation, transport, material, communication	Reports and documents from WHO consultants, EPI

COI: Costs of illness, CTC: Cholera treatment center, MoH: Ministry of Health of Zanzibar, NGO: Non-governmental organization, WHO HQ: World Health Organization headquarters, EPI: Expanded program on immunization in Zanzibar.

#### Public COI

Usually, CTCs are set up in Zanzibar once a cholera outbreak has been declared. Any identified person with acute watery diarrhea will be admitted and treated with IV fluids (Ringer's lactate, Hartmann's solution) and/or ORS, antibiotics and other drugs (Zinc for children) depending on the dehydration level. Community help-seeking behavior for cholera in peri-urban and rural Zanzibar also favors professional treatment in public health care facilities [**26**]. Thus, assuming that the majority of cases that occur during outbreaks are treated in CTCs, this study collected treatment costs incurred at CTCs to estimate public COI.

Public COI data from three outbreaks (one from Unguja and two from Pemba) that happened after the mass vaccination campaign were collected prospectively and retrospectively from local health care personnel and experts. All three centers were visited for an overview of how they were set up and being run. Fixed COI related to set up and running of centers, but considered independent of the number of cholera cases, included permanent material, consumables, transportation and personnel. The latter included extra (i.e., top up) payments for personnel and opportunity costs based on functions and salaries of personnel diverted from other health services. Variable COI incurred for cholera cases included drugs (resource use obtained from patient interviews) and material used for patient treatment. Current unit costs for drugs and material were provided by the chief pharmacist and the medical store department.

In Unguja, a CTC was opened on September 22, 2009, in PHCU Chumbuni after a cholera outbreak had been declared in one of the districts where the mass vaccination was conducted. A total of 161 patients were admitted over the course of 63 days before the CTC was closed on November, 29, 2009. Patients were treated in military tents (at the beginning of the outbreak) and in premises belonging to the PHCU. During the period while the CTC was operational, only suspected cholera cases were treated; patients with other illnesses were sent to adjacent clinics.

The first outbreak on Pemba occurred in Wete district, which is located between Micheweni district in the north and the Pemban capital Chake-Chake in the center of the island; the PHCU in Kiuyu Minungwini was turned into a CTC during 88 days from May 11, 2009, until August 7, 2009, when 88 patients were admitted and treated. The second outbreak on Pemba happened in Micheweni district in the northeast of the island. A school adjacent to PHCC Micheweni was turned into a CTC with male and female and pediatric wards. This center was first open from June 18, 2009, until August 11, 2009, to admit 349 patients over the course of 54 days. After another surge in cholera cases, the center was reopened on August 30, 2009, and run for an additional 31 days to treat another 32 patients until it was closed on September 30, 2009.

#### Private COI

Private COI data were collected with questionnaires from laboratory-confirmed cholera patients who had not received cholera vaccines before. A convenience sample of ∼100 respondents was selected based on a list of positive cases from outbreaks kept at the Public Health Laboratory (PHL) in Chake-Chake, Pemba. Health care providers were then contacted at the CTC where the patients had been admitted to confirm details and to contact the patient or the caregiver for an interview.

Based on WHO guidelines [27], a questionnaire was constructed in an adult and a child version to elicit out-of-pocket costs for cholera cases borne by patients and affected households. After pre-testing, the questionnaire was administered in face-to-face interviews to inquire about direct medical and non-medical costs and indirect costs (i.e., productivity losses to the patient or caregiver and other household members). Patients aged 18 years or older were directly interviewed while caregivers were interviewed if the patient was younger than 18 years. Questionnaires were administered between July and November 2009, predominantly at respondents' homes. Questionnaire data were entered into Microsoft Excel for analysis.

#### Mass Vaccination Campaign Costs

The mass vaccination campaign with Dukoral was implemented in the six selected Shehias in two rounds from January 17 to 26, 2009, and from February 7 to 16, 2009. Vaccination posts were erected within easy reach for the targeted population. Posts were run by local health care workers and villagers and open daily for at least eight hours. For each round, a total of 21 teams were needed to run the nine vaccination posts on each island. Each team consisted of six vaccinators. In addition, eight supervisors were deployed to Unguja and five to Pemba. The campaign was planned and implemented by the local Expanded Program on Immunization (EPI) team and international consultants deployed by the WHO. Social mobilization was done before and during both rounds by the MoH Health Promotion Unit.

Cost data on material (purchase, transport and storage of vaccines, cups and water), training and implementation required for the campaign were obtained locally from consultants and EPI. Because the campaign was planned and implemented within the scope of the research project, raw data were adjusted to exclude costs related to research. These costs were mostly incurred to train and compensate people at vaccination posts collecting data with electronic devices for parallel and subsequent epidemiological studies [28].

### Cost-Effectiveness Analysis

Based on a previous study for Bangladesh [29], a model was developed in Microsoft Excel to estimate the costs and health effects of a mass vaccination campaign program compared to standard treatment in CTCs in Zanzibar. A static cohort of 50,000 individuals, reflecting the target population of the 2009 mass vaccination campaign in Zanzibar, was examined from a health care provider and a societal perspective. Input parameters for inclusion in the model were related to vaccine characteristics and vaccination costs, burden and impact of cholera, and public and private COI. Private providers were not considered since the majority of patients would visit public facilities in case of an outbreak [26]. Indirect effects due to herd protection were also included in the model since they may play a considerable role in the overall impact of cholera vaccination [30] and were shown to make community-based programs in three Asian and one African setting cost-effective [15].

The base-case model considered costs and effects of a one-time vaccination program over the duration of protection (i.e., three years). The annual number of cases without vaccination was obtained by multiplying the population size times the annualized cholera incidence obtained from surveillance of diarrhea cases with laboratory confirmation for cholera in the study area [31]. The annual number of cases under the vaccination program was derived from adding up direct and indirect effects of the vaccination program: direct effects were calculated by multiplying the annual incidence of cases without vaccination with (1 – protective efficacy among vaccinated people [PE]), coverage, and population size; indirect effects were calculated by multiplying the annual incidence of cases without vaccination with (1 – protective efficacy among unvaccinated people [PEU]), (1 – coverage), and population size. The variable PEU was derived using the concepts and a formula from Longini *et al.* [30] (p. 1778). It calculates what they refer to as “indirect vaccine effectiveness” = 1−(r01/r02), where r01 is the cholera incidence among unvaccinated people within a vaccinated sub-region and r02 is the cholera incidence in an unvaccinated sub-region. In the absence of incidence data among unvaccinated people from the mass vaccination campaign area, the incidence rate of 2.34 cases per 1,000 population (after annualizing, Khatib *et al.* [31]) calculated from people that resided in the lowest quintile of surrounding coverage (i.e., <39%) in a cluster with a radius of 400 m around vaccinated households was used as proxy for r02. Khatib *et al.* showed that herd protection effects mainly existed within that radius. A longer distance from the household of the vaccinated person was considered to dilute the benefit of herd protection. The incidence of 1.29 cases per 1,000 population (after annualizing) among all unvaccinated people was used as an approximation for r01 [31]. This leads to a base-case estimate of PEU = 45%.

The number of annual deaths without a vaccination program was calculated by multiplying the CFR with the annual number of cases without vaccination. The number of deaths with a vaccination program was calculated by using the CFR times the annual number of cases under the vaccination program.

Incremental cost-effectiveness ratios (ICER) calculated as incremental costs per death, per case and per disability-adjusted life-year (DALY) averted were used as outcome measures. Incremental costs were calculated as the difference between costs of the vaccination program and public COI saved due to the vaccination from the health care provider perspective. Private direct COI saved were added in the base-case model adopting the societal perspective. Private indirect COI saved were not included in the base-case model [32]. The number of deaths, cases or DALYs averted was equal to the difference in numbers with and without the vaccination program. DALYs, which are an aggregate measure combining morbidity (i.e., years of life lived with disability) and mortality (years of life lost), were calculated according to Jeuland *et al.* [15], assuming no age weighing. Since no disability weights are available for cholera, the disability weight of 0.11 for diarrheal diseases [33] was used. Life expectancy at the average age of onset of 18 years based on patient data was obtained from WHO life tables for Tanzania [34]. The vaccine was directly purchased from the manufacturer at a UN price. Future effects were discounted at a rate of 3.0% for the base case. Campaign costs were not discounted since the mass campaign happened over one single year.

Cost-effectiveness was examined according to widely-used WHO criteria that define an intervention as ‘cost-effective’ if the ICER is less than three times per capita gross domestic product (GDP) per DALY averted and as ‘highly cost-effective’ if the ICER is less than per capita GDP per DALY averted [35].

One-way sensitivity analyses were done to estimate the influence of changes in potentially influential input parameters on model outcomes. Such key parameters included vaccine purchase price and delivery costs, protective efficacy (PE, PEU), duration of protection, incidence, CFR and so forth [15]. Plausible ranges were based on public health considerations (for vaccine purchase price and delivery costs, incidence), guidelines (discount rate) and variation for local data (PE, PEU, CFR, number of ill days, public and private COI). Base-case values and plausible ranges are presented in Table 2. Threshold analyses examined at which vaccine purchase price the intervention would become cost-effective.

**Table 2:** Model input parameters with plausible ranges.

Parameters	Base case	Minimum	Maximum	Assumptions, References
**Vaccine costs and characteristics**				
Vaccine purchase price, 2009 USD per 2 doses	10	2.1	12	Base case: this study; range: 20–120% of base case based on policymaker and expert data [36], [37]
Vaccine delivery, 2009 USD per 2 doses^a^	2.7	1.1	3.2	Base case: this study; range: from USD 0.5 per dose to 120% of base case [15], [37]
Protective efficacy among vaccinated people (PE), %	79	47	92	Base case and range (95% CI) [31]
Protective efficacy among unvaccinated people (PEU), %	45	0.0	75	Base case and maximum [31]; minimum: assuming no indirect protection
Campaign coverage, %	50	NA	NA	Khatib *et al.* [31]
Duration of protection, years	3.0	2.0	4.0	Jeuland *et al.* [15]
Discount rate, %	3.0	0.0	5.0	Constant, for effects [38], no discounting of costs
Life expectancy at average age of onset, years	45	36	56	Life tables for WHO member states [34]; base case: based on mean age of onset (18 years) from patient data; range: based on life expectancy [34] at IQR of age of onset from patient data
**Risk for cholera**				
Cholera incidence, annual cases per 1,000 population	2.3	0.50	4.0	Base case [31]; range: minimum (Jakarta), maximum (Beira) [39]
**Impact of illness on patients**				
Case-fatality rate, %	0.86	0.52	1.9	Base case: 14 deaths/1626 cases treated in CTCs in Unguja and Pemba during three outbreaks between June 2009 and April 2010; range: minimum and maximum (ZMO Unguja); same rate assumed for vaccinated and unvaccinated cases
Duration of illness episode, days	5	4	6	Base case: median illness duration from patient data; range: IQR from patient data
**Costs of illness, 2009 USD**				
Public fixed costs of treatment per episode	51	21	88	Base case: mean from this study (see Table 3); range: minimum and maximum from this study (see Table 3)
Public variable costs of treatment per episode	9.2	4.6	18	Base case: mean from this study (see Table 3); range: 50–200% of base case [15]
Private direct costs per episode^b^	11	4.2	17	Base case: mean from this study (see Table 4); range: based on IQR from patient data
Private indirect costs per episode^b^	32	4.4	46	Base case: mean from this study (see Table 4); range: based on IQR from patient data

a Excluding costs for international consultants (see Table 5);

b Estimates only used in analysis from societal perspective;

CI: Confidence intervals, IQR: Interquartile range, ZMO: Zonal medical officer.

## Results

### Public COI

Table 3 presents the fixed and variable mean public COI at the three CTC sites. Fixed costs of USD 51 accounted for 85% of public COI, with mean fixed costs ranging from USD 21 to USD 88. Direct and indirect human resources costs accounted for the majority of fixed costs; they were highest in Kiuyu Minungwini (86%), medium in Micheweni (85%) and lowest in Chumbuni (80%). The remaining fixed costs were used for setting up and running the centers. Health care personnel working in Unguja received higher top up payments than in Pemba, but the latter were given food to cater for themselves while on shift. Variable costs of USD 9.2 were mainly driven by IV fluid use as patients were administered on average 8.8 liters, which cost USD 7.1. Further details on public variable costs for treatment can be found as supporting information in Table S1.

**Table 3:** Public costs of illness for cholera, Zanzibar, 2009.

	Description	2009 USD	%
**Fixed costs** a		**51**	**85**
CTC at PHCU Chumbuni (Unguja)		88	100
*Permanent material*	*Beds, canvas, ropes, basins, buckets, further utensils*	*6.4*	*7.2*
*Consumables*	*Water, detergent, kerosene*	*2.5*	*2.9*
*Transport*	*Fuel for DHMT cars*	*8.7*	*9.9*
*Personnel*	*Top up payments*	*27*	*31*
*Personnel diverted from other health care services*	*Opportunity costs based on functions and official salaries of health care workers deployed to CTCs*	*43*	*49*
CTC at PHCC Micheweni (Pemba)		21	100
*Permanent material*	*Water drum*	*0.0*	*0.0*
*Consumables*	*Detergent, kerosene*	*1.2*	*5.6*
*Transport*	*Fuel for DHMT cars*	*1.9*	*9.4*
*Personnel*	*Top up payments and food allowance*	*6.0*	*29*
*Personnel diverted from other health care services*	*Opportunity costs based on functions and official salaries of health care workers deployed to CTCs*	*12*	*56*
CTC at PHCU Kiuyu Minungwini (Pemba)		46	100
*Permanent material*	*Water tank, cooking utensils etc*	*1.9*	*4.1*
*Consumables*	*Chlorinated lime*	*1.7*	*3.8*
*Transport*	*Car use*	*2.6*	*5.8*
*Personnel*	*Top up payments and food allowance*	*14*	*30*
*Personnel diverted from other health care services*	*Opportunity costs based on functions and official salaries of health care workers deployed to CTCs*	*26*	*57*
**Variable costs** b		**9.2**	**15**
**Total costs**		**61**	**100**

a Mean costs per treated patient at each CTC;

b Mean costs per treated patient from patient interviews (n = 95), including drugs and material, see supporting information (Table S1) for more details;

CTC: Cholera treatment center, PHCU: Primary health care unit, PHCC: Primary health care center, DHMT: District health management team.

### Private COI

A total of 95 individuals were interviewed. All but one of the interviewed patients had been admitted at the CTC at Micheweni PHCC. Total direct and indirect mean private COI amounted to USD 43, with almost three-fourth (USD 32) being indirect costs (i.e., productivity losses to the patient or caregiver and other household members) (Table 4). Among direct costs, which amounted to USD 11, feeding the patient at the CTC accounted for the biggest share (USD 8.3, 19% of total costs). Other direct costs, incurred for treatment (mainly for plastic sheets needed to cover cots), transport and communication, were reported each by less than 3%.

**Table 4:** Private direct and indirect costs of illness for cholera, Zanzibar, 2009.

	2009 USD^a^		%
**Direct costs**	**11**	**(9.1)**	**27**
Medical	1.2	(1.6)	2.8
Food	8.3	(6.6)	19
Transport	1.2	(2.7)	2.9
Communication	0.65	(1.4)	1.5
**Indirect costs (i.e., lost productivity)**	**32**	**(35)**	**73**
**Total costs**	**43**	**(40)**	**100**

a Mean costs (standard deviation in brackets) per treated patient from patient interviews (n = 95).

### Mass Vaccination Campaign Costs

Total mass vaccination campaign costs amounted to USD 760,000, with USD 510,000 (68%) spent on vaccine purchase and USD 240,000 (32%) on delivery (Table 5). The vaccine was purchased from SBL Vaccin AB, Sweden, at a price of USD 10 per course (2 doses). Delivery costs comprised transport of the vaccine from Stockholm to Zanzibar and procurement of cups and water required for the buffer solution (6.0% of campaign costs), the work of two experienced international consultants (14%), training of locally recruited implementers (1.3%) and the implementation (social mobilization and vaccination) itself (10%). More details on delivery costs are presented as supporting information in Table S2.

**Table 5:** Costs of a mass oral cholera vaccination campaign, Zanzibar, 2009.

	Total^a^	Mean^b^	%
**Vaccine** (purchase price USD 10 per course)	**510,000**	**21**	**68**
**Delivery** c	**240,000**	**9.7**	**32**
Vaccine transport, storage, water and cups	45,000	1.8	6.0
International consultants	110,000	4.4	14
Training	9,500	0.38	1.3
Implementation	78,000	3.2	10
**Total costs**	**760,000**	**30**	**100**

a Total costs (2009 USD) to vaccinate a target population of 49,980 people;

b Mean costs (2009 USD) per fully immunized individual based on actual coverage (50%);

c Based on actual expenditure or planned budget data from 2009 mass vaccination campaign, see supporting information (Table S2) for more details.

At a vaccine purchase price of USD 10 per course, the estimated total costs per fully immunized individual amounted to USD 30, with mean costs per vaccine course of USD 21 and mean costs for delivery of USD 9.7. The latter amounted to USD 5.3 after exclusion of services from international consultants, lowering the overall total costs per fully immunized individual to USD 26. Mean costs were adjusted for actual coverage of 50%, relating to 23,921 fully immunized individuals out of a population denominator of 48,178 used in the analysis by Khatib *et al.* [31].

### Cost-Effectiveness Analysis

#### Base-Case Results

Table 6 presents the results of the cost-effectiveness analysis from the health care provider perspective using base-case parameter estimates obtained from primary and secondary data sources from Zanzibar. Annual costs to immunize 50,000 people, if the OCVs cost USD 10 per course, were USD 430,000 assuming one campaign per three years at a cost of USD 1.3 million. Annual public COI averted by vaccination amounted to USD 4,000. Incremental costs, the difference between total annual costs (i.e., vaccination program and public COI) with and without vaccination, amounted to USD 430,000. ICERs were USD 750,000 per death averted, USD 6,500 per case averted and USD 30,000 per DALY averted.

**Table 6:** Key outcomes from model of mass oral cholera vaccination (health care provider perspective) in Zanzibar, 2009.

	No vaccination	Vaccination	Difference
**Effects**			
Annual number of cases	110	41	69
Annual number of deaths	0.92	0.35	0.57
Annual number of YLD averted			0.09
Annual number of YLL averted			14
Annual number of DALY averted			14
Total number of DALY averted over duration of protection			40
**Costs of outcome indicators, 2009 USD**			
Annual costs of vaccination program^a^	0	430,000	−430,000
Annual public costs of illness	6,500	2,500	4,000
Annual costs of treatment and vaccination program	6,500	440,000	−430,000
Costs per death averted with vaccine		760,000	
Costs per case averted with vaccine		6,600	
Costs per DALY averted with vaccine		31,000	
**Incremental costs and cost-effectiveness ratios (ICER), 2009 USD**			
Incremental costs^b^			430,000
ICER (death): Incremental costs/death averted			750,000
ICER (case): Incremental costs/case averted			6,500
ICER (DALY): Incremental costs/DALY averted			30,000

Base-case results from population of 50,000, with 3% annual discounting of effects.

a Costs for international consultants excluded;

b Costs of vaccination program minus public COI averted by vaccination (cost savings);

YLD: Years of life lived with disability, YLL: Years of life lost, DALY: Disability-adjusted life-year, ICER: Incremental cost-effectiveness ratio.

Logistical on-site support from the WHO headquarters was provided since the campaign was conducted within a research project that aimed to assess also epidemiological and socio-behavioral aspects of OCV use in endemic settings. Thus, costs incurred for international consultants were excluded from the analysis on the assumption that the campaign would also have been possible without intensive external help.

The predicted ICER was much greater than three times the per capita GDP for Tanzania (USD 1,500 in 2009) per DALY averted [40], suggesting that mass immunization with OCV in Zanzibar was not cost-effective. Even if the OCV was donated to the government at no cost, the vaccination would still cost more than the avoided public COI due to the delivery costs; and the ICER would be USD 6,000 per DALY averted.

Compared to the health care provider perspective, key outcomes of the cost-effectiveness analysis from the societal perspective (Table S3), which included private direct COI, did not differ from each other.

#### Sensitivity Analyses

One-way sensitivity analyses from the health care provider perspective were performed with input parameters presented in Table 2. This analysis does not account for the effects of non-linearity and interactions between uncertain parameters as it varies parameters one at-a-time while keeping other parameters at base-case values, and the ranges specified for each parameter may not reflect equivalent ranges of uncertainty [41]. Varying base-case values over plausible ranges helped to estimate the influence of parameters on the ICER per DALY averted (see Figure S1), and per death (Figure S2) and case (Figure S3) averted. The most influential parameters on the ICER per DALY averted were incidence, CFR, discount rate and vaccine purchase price.

In the absence of herd protection (i.e., if PEU = 0%), the ICER per DALY amounted to USD 48,000; and when assuming a herd protection rate as achieved in Zanzibar (PEU = 75%), the ICER of USD 24,000 still by far exceeded the criterion of USD 1,500, below which the intervention was defined as cost-effective.

Acknowledging that the incidence would have been higher than the base-case estimate used in the model if there had been no OCV campaign at all in Zanzibar, an annual incidence of 4.0 cases per 1,000 population from Beira was used as an upper bound in the sensitivity analysis. Even though the ICER was reduced to USD 18,000 per DALY averted at this incidence rate, the campaign was still cost-ineffective.

#### Threshold Analyses

Another two-dose OCV was licensed for use in India in 2009. Shanchol (Shanta Biotechnics, Hyderabad, India) is a bivalent variant of Dukoral, containing killed *V. cholerae* O1 and O139, but no CT B subunit. It has recently been pre-qualified by the WHO for UN use; at its current price of USD 1.9 per dose to the public sector, it may become an attractive alternative for future OCV campaigns [22]. Analysis from three years of follow-up of a randomized controlled trial from Kolkata, India, showed that Shanchol has a protective efficacy (PE) of 66% across all age groups [42]. Repetition of the OCV campaign in Zanzibar with Shanchol at USD 3.7 per course and PE = 66%—*ceteris paribus*—would reduce the ICER to USD 16,000 per DALY averted from the health care provider and the societal perspectives.

In addition, changing vaccine delivery costs to USD 1.1 (being the minimum level used in this study), cholera incidence to 4.0 cases per 1,000 population and CFR to 1.9% (both parameters being at the maximum level) would further reduce the ICER to USD 3,300 per DALY averted from both perspectives. Based on these assumptions the purchase price of Shanchol per course would have to be as low as USD 1.2 to render the mass vaccination campaign cost-effective from a health care provider perspective (or USD 1.3 from a societal perspective).

## Discussion

This study estimated public and private COI due to endemic cholera in Zanzibar and costs of the 2009 mass vaccination campaign to assess cost-effectiveness from a health care provider and a societal perspective. The analysis presented here suggests that COI averted by a mass vaccination campaign with an OCV were negligible to the public health sector and the society and that such an intervention was not cost-effective based on the stated assumptions. However, mass vaccination campaigns in Zanzibar to control endemic cholera may meet WHO criteria for cost-effectiveness under certain circumstances of highly optimistic assumptions about vaccine purchase price, delivery costs, incidence and CFR. It should also be noted that the ICERs do not explicitly account for the indirect COI and the societal value of prevented premature deaths, which are implicitly captured in the effectiveness in terms of averted DALYs.

Private costs were higher than in Beira, Mozambique [13], where Dukoral had also been used in a mass campaign in endemic settings. Although mean public and private COI of USD 104 per episode were higher than the USD 47 for hospitalized cases in Beira, the mass vaccination campaign was not cost-effective in Zanzibar.

Relative costs for the vaccine and for delivery were comparable to findings from two campaigns in Vietnam with the bivalent Vietnamese OCV where this ratio was 25 vs. 75% in 1997 [43] and 21 vs. 79% in 1998 [12], respectively. However, due to the high vaccine purchase price, mean costs per fully immunized individual of USD 21 were much higher than previously reported costs of USD 0.5 to 10 from Sudanese refugee settlements in northern Uganda (1997) [44] and of USD 2.1 from Beira, Mozambique (2003), where the vaccine was provided free of charge [14].

Mean costs for delivery of USD 5.3 (after exclusion of costs related to international consultants) tended to be more in the range of previous campaigns in other regions; costs in the Zanzibar campaign were between the USD 3.3 reported for Darfur, Sudan (2004) [45], and the USD 8.6 reported for the mass immunization campaign in post-tsunami Aceh, Indonesia (2005) [46].

ICERs were well above any results reported for previous cholera mass vaccination campaigns [15], [29], [47]–[50]. The main reason why mass vaccination with Dukoral was cost-ineffective in Zanzibar may be due to using an expensive OCV in a relatively low incidence setting. Another reason may be that the present model used local data on costs of immunization. Other cost-effectiveness models that were not based on locally available data generally assumed much lower immunization costs, using (subsidized) vaccine prices of ∼USD 1 and delivery costs of ∼USD 1 per course; this made them propose that vaccination is economically more viable than standard treatment [15].

Also noteworthy in contrast to Jeuland *et al.* [15] is the finding that the inclusion of indirect effects in the model did not make the intervention cost-effective. The ICERs per DALY averted for both the base-case and the maximum estimate for herd protection were well above the WHO criterion for cost-effectiveness.

### Limitations

This study has several limitations. First, due to limited data availability, this cost-effectiveness analysis assessed the value for money of a population-wide OCV campaign and not of a targeted approach for high-risk or specific age groups, which might make the intervention cost-effective as shown by Jeuland *et al.* for school-based programs in Kolkata and Beira [15]. However, threshold analyses using Shanchol indicated that scenarios targeting high-risk groups may become cost-effective in Zanzibar if the OCV was procured at a price below USD 1.3, a level acceptable by many public health policy makers in Asia [36].

Second, it may be argued that the assumption of a preference for health facilities during a cholera outbreak may not necessarily reflect actual behavior as patients could also be negatively influenced by accessibility problems. Local observation and informal interviews, however, support this assumption, and the dense primary health care system reduces transport issues (according to the 2004/5 household budget survey, mean distance to a health care center in the urban and rural area was 0.4 and 1.7 km, respectively [17]) and because treatment for diarrhea is free.

Third, non-diarrhea patients were usually not treated or admitted by their local public health care facility during the time it operated as a CTC. People seeking treatment for non-diarrheal diseases (e.g., for malaria) during an ongoing cholera outbreak will have to bear extra direct and indirect costs related to additional travel or potential serious complications due to delayed treatment. These additional costs have not been included in the cost-effectiveness analysis due to a lack of relevant data; future studies in the area are advised to collect estimates on the costs of patients who are not able to get treatment at their usual center to assess the relevance of this ‘crowding out’ effect on cost-effectiveness.

Fourth, the ICER might have been overestimated because waning has not been included in the estimate for PE. Jeuland *et al.* have adjusted their estimate in year 3 down by 17% [15]. However, since this represents a limited effect, and since sensitivity analysis included a minimum PE of 47%, omission of waning as input parameter in the model is likely to have only a limited effect.

Fifth, threshold analysis for Shanchol did not consider potential savings due to the probably easier and faster administration of this new vaccine by oral syringe; this may have resulted in more favorable cost-effectiveness, but any beneficial effect will likely be limited because delivery costs influenced the ICER only to a small extent.

Sixth, even though uncertainty in input parameters was considered in one-way sensitivity analyses, no full probabilistic uncertainty and sensitivity analysis was conducted which would provide a more complete picture of the distribution of possible outcomes and may find that some combinations of assumptions lead to greater cost-effectiveness than identified in the one-way sensitivity analysis [38].

Finally, a cost-benefit analysis may provide more useful information to local policy makers than a cost-effectiveness analysis because it explicitly characterizes the monetary value of prevented disease. However, willingness-to-pay data were not available for Zanzibar and contingent valuation exercises were beyond the scope of this study. Also, since campaign coverage with the free OCV in Zanzibar was merely 50% overall [31] and the community demanded a free vaccine [51], not making the OCV available for free in future campaigns would further jeopardize vaccine effectiveness and thus make such a program even less economical.

### Conclusions

The analysis presented here suggests that costs averted by a mass vaccination campaign with an OCV in endemic areas of Zanzibar were negligible when compared to standard treatment in decentralized cholera treatment centers. Mass vaccination was not cost-effective based on empirical data and the stated assumptions, mainly due to the relatively high purchase price and the relatively low cholera incidence in Zanzibar. However, mass vaccination campaigns in Zanzibar for endemic cholera control may meet WHO criteria for cost-effectiveness under certain circumstances, especially in high-incidence areas and when OCV prices are reduced to levels below USD 1.3.

## Supporting Information

Figure S1 **One-way sensitivity analysis of the influence of key parameters on ICER in 2009 USD per DALY averted from model of mass oral cholera vaccination (health care provider perspective) in Zanzibar, 2009.** Tornado diagram presents parameters that were varied over their plausible ranges, as shown in brackets. Vertical line indicates base-case ICER of USD 30,000 per DALY averted. ICER: Incremental cost-effectiveness ratio, DALY: Disability-adjusted life-year.
(PDF)

Figure S2 **One-way sensitivity analysis of the influence of key parameters on ICER in 2009 USD per death averted from model of mass oral cholera vaccination (health care provider perspective) in Zanzibar, 2009.** Tornado diagram presents parameters that were varied over their plausible ranges, as shown in brackets. Vertical line indicates base-case ICER of USD 750,000 per death averted. ICER: Incremental cost-effectiveness ratio.
(PDF)

Figure S3 **One-way sensitivity analysis of the influence of key parameters on ICER in 2009 USD per case averted from model of mass oral cholera vaccination (health care provider perspective) in Zanzibar, 2009.** Tornado diagram presents parameters that were varied over their plausible ranges, as shown in brackets. Vertical line indicates base-case ICER of USD 6,500 per case averted. ICER: Incremental cost-effectiveness ratio.
(PDF)

Table S1 **Public variable costs of illness for cholera, Zanzibar, 2009.**
(PDF)

Table S2 **Delivery costs for a mass oral cholera vaccination campaign, Zanzibar, 2009.**
(PDF)

Table S3 **Key outcomes from model of mass oral cholera vaccination (societal perspective) in Zanzibar, 2009.** Base-case results from population of 50,000, with 3% annual discounting of effects.
(PDF)
