# Supplementary figures and images for: Costs of Illness Due to Cholera, Costs of Immunization and Cost-Effectiveness of an Oral Cholera Mass Vaccination Campaign in Zanzibar
Source: PLoS Negl Trop Dis. 2012 Oct 4;6(10):e1844. doi: 10.1371/journal.pntd.0001844 (PMC3464297; doi:10.1371/journal.pntd.0001844)

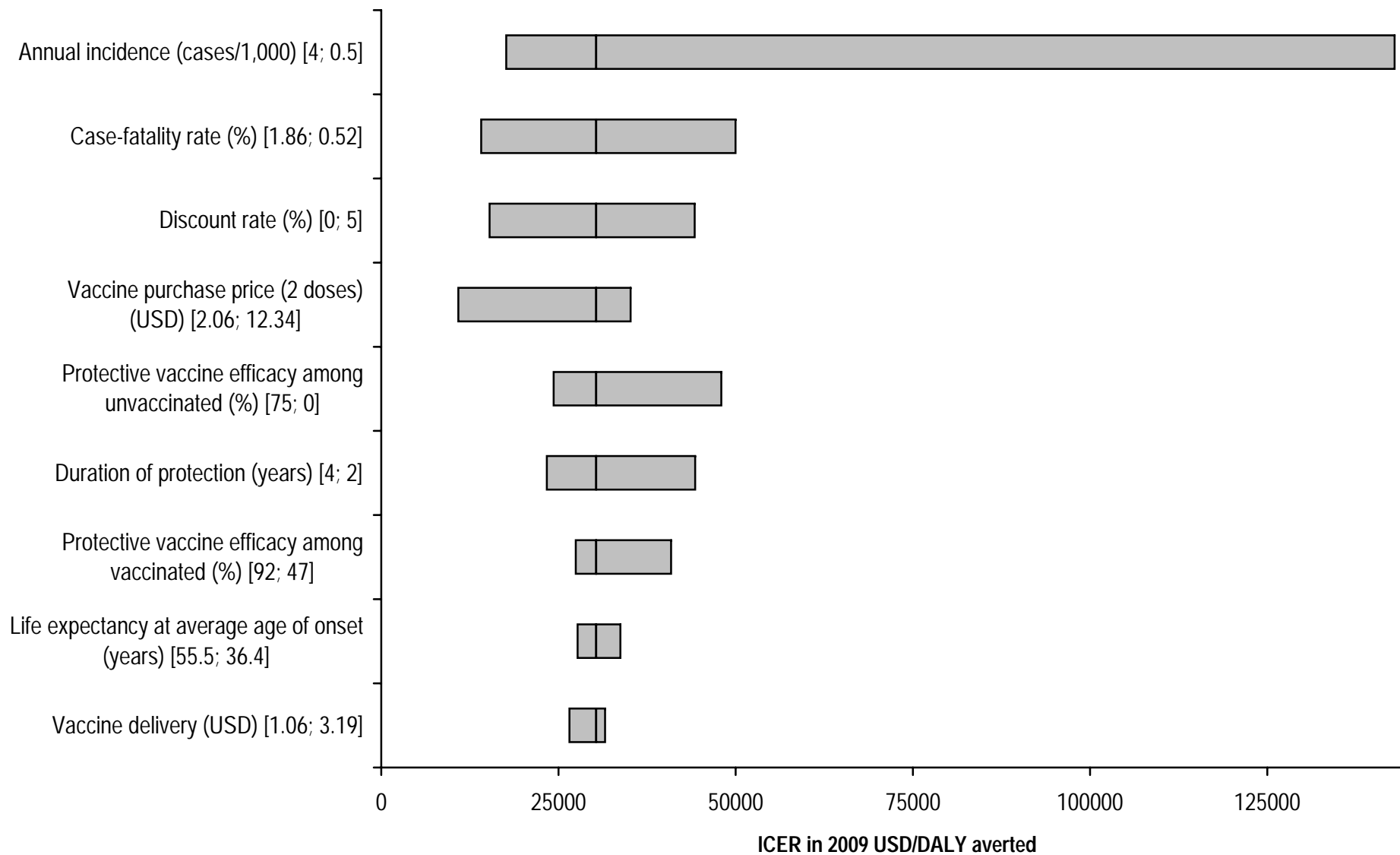

Supplement: Figure S1 — One-way sensitivity analysis of the influence of key parameters on ICER in 2009 USD per DALY averted from model of mass oral cholera vaccination (health care provider perspective) in Zanzibar, 2009. Tornado diagram presents parameters that were varied over their plausible ranges, as shown in brackets. Vertical line indicates base-case ICER of USD 30,000 per DALY averted. ICER: Incremental cost-effectiveness ratio, DALY: Disability-adjusted life-year. (PDF) [file pntd.0001844.s001.pdf]

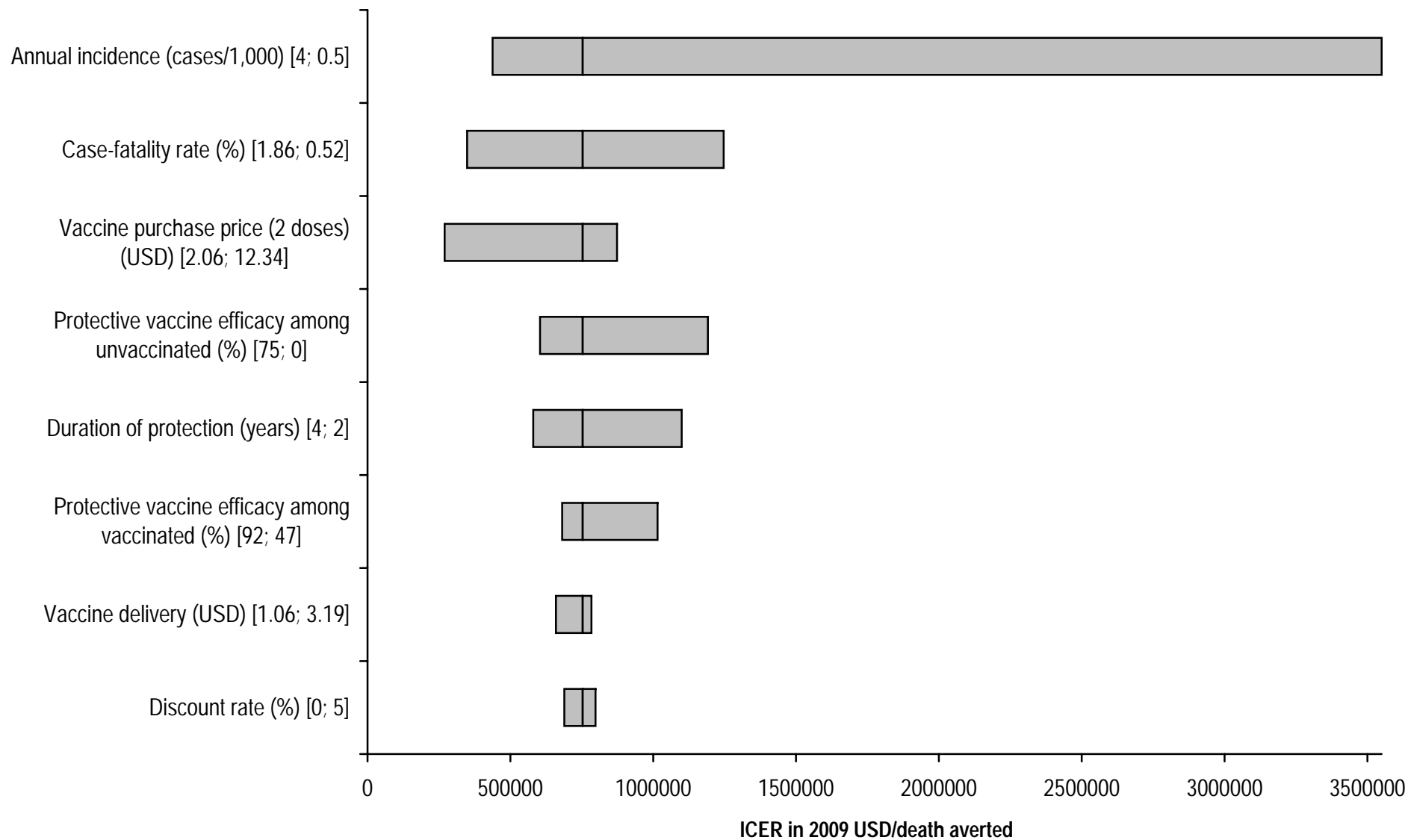

Supplement: Figure S2 — One-way sensitivity analysis of the influence of key parameters on ICER in 2009 USD per death averted from model of mass oral cholera vaccination (health care provider perspective) in Zanzibar, 2009. Tornado diagram presents parameters that were varied over their plausible ranges, as shown in brackets. Vertical line indicates base-case ICER of USD 750,000 per death averted. ICER: Incremental cost-effectiveness ratio. (PDF) [file pntd.0001844.s002.pdf]

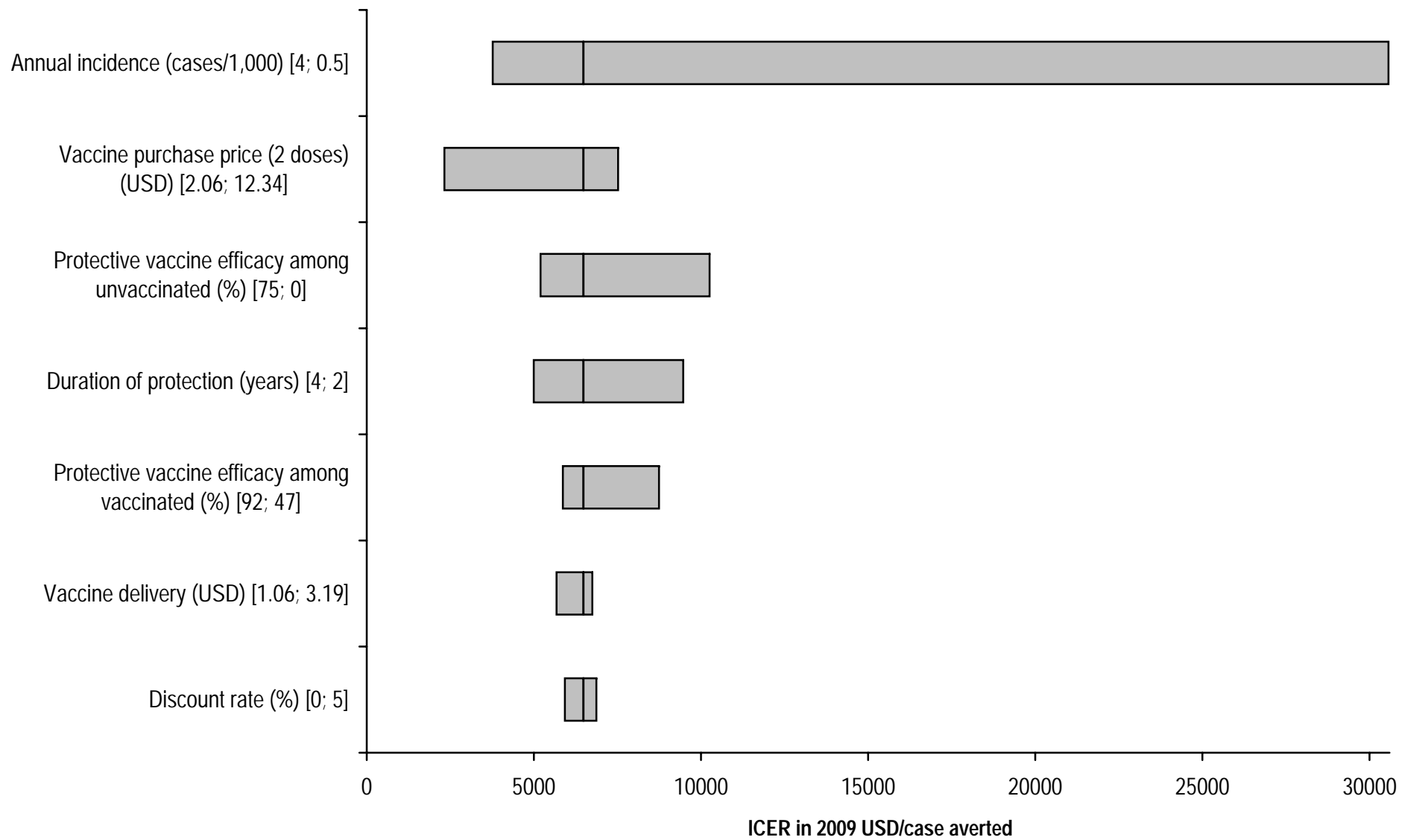

Supplement: Figure S3 — One-way sensitivity analysis of the influence of key parameters on ICER in 2009 USD per case averted from model of mass oral cholera vaccination (health care provider perspective) in Zanzibar, 2009. Tornado diagram presents parameters that were varied over their plausible ranges, as shown in brackets. Vertical line indicates base-case ICER of USD 6,500 per case averted. ICER: Incremental cost-effectiveness ratio. (PDF) [file pntd.0001844.s003.pdf]
