# Supplementary material for: Costs of Illness Due to Cholera, Costs of Immunization and Cost-Effectiveness of an Oral Cholera Mass Vaccination Campaign in Zanzibar
Source: PLoS Negl Trop Dis. 2012 Oct 4;6(10):e1844. doi: 10.1371/journal.pntd.0001844 (PMC3464297; doi:10.1371/journal.pntd.0001844)
Supplement: Table S1 — Public variable costs of illness for cholera, Zanzibar, 2009. (PDF) [file pntd.0001844.s004.pdf]

Table S1. Public variable costs of illness for cholera, Zanzibar, 2009.

	2009 USD[a]		%
Drugs[b]	8.0	(7.9)	88
Antibiotic: Ciproxine	0.01	(0.08)	0.1
Antibiotic: Doxycycline	0.01	(0.13)	0.1
Antibiotic: Erythromycine	0.52	(0.41)	5.6
Antibiotic: Erythromycine syrup	0.10	(0.48)	1.1
Antibiotic: Metronidazole	0.01	(0.04)	0.2
Antibiotic: Septrine	0.01	(0.04)	0.1
IV fluid	7.1	(7.7)	77
Oral rehydration solution	0.25	(0.14)	2.8
Other drugs: Mebendazole (antihelminthic)	0.01	(0.01)	0.1
Other drugs: Zinc sulphate	0.03	(0.07)	0.3
Material[c]	1.1	(0.0)	12
Cannula (adults)	0.34	(0.0)	3.8
Examination gloves	0.52	(0.0)	5.7
IV giving set	0.20	(0.0)	2.1
Zinc oxide plaster	0.05	(0.0)	0.6
Total costs	9.2	(7.9)	100

[a]Mean costs and standard deviation in brackets;
[b]Drug resource use based on patient interviews (n = 95), drug unit costs include 6.0% for storage at medical store department and distribution;
[c]Standard resource use per patient based on expert interview: cannula (1-2 pieces), examination gloves (8 pairs), IV giving sets (1 piece) and Zinc oxide plaster (30 cm);
IV: Intravenous.
