# Supplementary material for: Costs of Illness Due to Cholera, Costs of Immunization and Cost-Effectiveness of an Oral Cholera Mass Vaccination Campaign in Zanzibar
Source: PLoS Negl Trop Dis. 2012 Oct 4;6(10):e1844. doi: 10.1371/journal.pntd.0001844 (PMC3464297; doi:10.1371/journal.pntd.0001844)
Supplement: Table S2 — Delivery costs for a mass oral cholera vaccination campaign, Zanzibar, 2009. (PDF) [file pntd.0001844.s005.pdf]

Table S2. Delivery costs for a mass oral cholera vaccination campaign, Zanzibar, 2009.

	Total[a]	Mean[b]	%
Vaccine transport, storage, water and cups	45,000	1.8	19
International transport of vaccine[c]	20,000	0.80	8.2
Purchase of cups and international transport[d]	9,300	0.38	3.9
Purchase of bottled water[e]	4,600	0.19	1.9
Storage and local transport of vaccines, cups and water[f]	11,000	0.46	4.7
International consultants[g]	110,000	4.4	45
Compensation, travel	110,000	4.4	45
Training	9,500	0.38	3.9
Training vaccinators	5,800	0.23	2.4
Training social mobilizers	3,700	0.15	1.5
Staff allowances	*2,100*	*0.08*	*0.9*
Staff transport	*290*	*0.01*	*0.1*
Material	*640*	*0.03*	*0.3*
Refreshment	*530*	*0.02*	*0.2*
Venue	*110*	*0.00*	*0.0*
Implementation	78,000	3.2	32
Vaccination	66,000	2.7	27
Staff allowances	*33,000*	*1.3*	*14*
Staff transport	*7,100*	*0.29*	*2.9*
Material	*26,000*	*1.0*	*11*
Communication	*350*	*0.01*	*0.1*
Social mobilization[h]	12,000	0.49	5.1
Total costs	240,000	9.7	100

[a]Total delivery costs (2009 USD) to vaccinate a target population of 49,980 people;
[b]Mean delivery costs (2009 USD) per fully immunized individual based on actual coverage (50%);
[c]Vaccine transported from Stockholm, Sweden;
[d]Disposable paper cups purchased at a price of 8 US cents and transported from Shanghai, China;
[e]15,000 liters of drinking water procured from a local agent in 1.5 liter plastic bottles at TZS 600 (USD 0.5) per bottle;
[f]Costs for storage of vaccines, cups and water at the medical store department including cold room facilities and generator maintenance and transport to vaccination posts;
[g]Involvement of two international consultants;
[h]Includes material (T-shirts, posters, leaflets, banners, radio/TV programs) and staff costs.
