# Supplementary material for: Costs of Illness Due to Cholera, Costs of Immunization and Cost-Effectiveness of an Oral Cholera Mass Vaccination Campaign in Zanzibar
Source: PLoS Negl Trop Dis. 2012 Oct 4;6(10):e1844. doi: 10.1371/journal.pntd.0001844 (PMC3464297; doi:10.1371/journal.pntd.0001844)
Supplement: Table S3 — Key outcomes from model of mass oral cholera vaccination (societal perspective) in Zanzibar, 2009. Base-case results from population of 50,000, with 3% annual discounting of effects. (PDF) [file pntd.0001844.s006.pdf]

Table S3. Key outcomes from model of mass oral cholera vaccination (societal perspective) in Zanzibar, 2009.

	No vaccination	Vaccination	Difference
**Effects**			
Annual number of cases	110	41	69
Annual number of deaths	0.92	0.35	0.57
Annual number of YLD averted			0.09
Annual number of YLL averted			14
Annual number of DALY averted			14
Total number of DALY averted over duration of protection			40
**Costs of outcome indicators, 2009 USD**			
Annual costs of vaccination program[a]	0	430,000	-430,000
Annual public costs of illness	6,500	2,500	4,000
Private costs of illness (direct)	1,200	460	760
Annual costs of treatment and vaccination program	7,700	440,000	-430,000
Costs per death averted with vaccine		760,000	
Costs per case averted with vaccine		6,600	
Costs per DALY averted with vaccine		31,000	
**Incremental costs and cost-effectiveness ratios (ICER), 2009 USD**			
Incremental costs[b]			430,000
ICER (death): Incremental costs/death averted			750,000
ICER (case): Incremental costs/case averted			6,500
ICER (DALY): Incremental costs/DALY averted			30,000

Base-case results from population of 50,000, with 3% annual discounting of effects.
[a]Excluding costs for international consultants;
[b]Costs of vaccination program minus public and private COI averted by vaccination (cost savings);
YLD: Years of life lived with disability, YLL: Years of life lost, DALY: Disability-adjusted life-year, ICER: Incremental cost-effectiveness ratio.
